# Healthcare efficiency across GCC countries: an exploratory DEA study

**DOI:** 10.3389/fpubh.2026.1877020

**Published:** 2026-07-07

**Authors:** Majed Ahmed Algarni

**Affiliations:** Department of Clinical Pharmacy, College of Pharmacy, Taif University, Taif, Saudi Arabia

**Keywords:** data envelopment analysis, efficiency, Gulf cooperation council, health policy, healthcare expenditure, life expectancy

## Abstract

**Background:**

Healthcare systems in the Gulf Cooperation Council (GCC) countries have expanded substantially in recent decades, accompanied by increasing healthcare expenditures. However, higher spending does not necessarily translate into proportionally better population health outcomes, highlighting the importance of evaluating healthcare expenditure efficiency.

**Objective:**

This exploratory study assessed the relative efficiency of healthcare expenditure across GCC countries using Data Envelopment Analysis (DEA).

**Methods:**

An exploratory cross-sectional comparative analysis was conducted across six GCC countries: Saudi Arabia, United Arab Emirates, Qatar, Kuwait, Bahrain, and Oman. Health expenditure per capita, expressed in purchasing power parity (PPP)-adjusted current international USD, was used as the input variable, while life expectancy at birth was used as the output variable. Data were obtained from the World Bank World Development Indicators and World Health Organization databases. An input-oriented variable returns to scale (VRS) DEA model was applied. Sensitivity analyses were conducted using alternative DEA specifications, including constant returns to scale (CRS), input-oriented, and output-oriented models.

**Results:**

Relative healthcare expenditure efficiency varied across GCC countries. Qatar and Oman were positioned on the efficiency frontier under the primary VRS model, whereas Bahrain and Kuwait showed relatively high efficiency scores. The United Arab Emirates and Saudi Arabia demonstrated comparatively lower scores. Sensitivity analyses produced broadly consistent findings. However, the results should be interpreted cautiously because of the exploratory design, limited number of decision-making units, and dependence of DEA estimates on model specification. DEA efficiency scores represent relative efficiency within the selected framework and should not be interpreted as direct measures of healthcare quality or overall health system performance.

**Conclusion:**

Healthcare expenditure efficiency varied across GCC countries, suggesting that higher spending does not consistently correspond to better population health outcomes. These preliminary findings emphasize the importance of effective resource utilization. Future research using larger datasets, longitudinal designs, and broader health system indicators is needed.

## 1 Introduction

Health systems globally are under increasing pressure to deliver improved population health outcomes while containing rising healthcare expenditures. Although higher levels of health spending are often assumed to lead to better health outcomes, empirical evidence suggests that the relationship is neither linear nor guaranteed. Instead, the efficiency with which healthcare resources are allocated and utilized plays a decisive role in determining system performance (1–3).

The countries of the Gulf Cooperation Council (GCC)—including Saudi Arabia, United Arab Emirates, Qatar, Kuwait, Bahrain, and Oman—have experienced rapid economic growth and substantial expansion in healthcare infrastructure over recent decades. According to the World Bank and the World Health Organization, healthcare expenditure in GCC countries has increased significantly, positioning several of them among the highest spenders in the Middle East and North Africa (MENA) region (4, 5). Despite these investments, health outcomes—particularly those related to NCDs—remain a major public health concern.

Non-communicable diseases, including cardiovascular diseases, diabetes mellitus, and chronic respiratory conditions, account for the majority of morbidity and mortality in GCC countries. The region is undergoing an advanced epidemiological transition characterized by high prevalence of metabolic risk factors such as obesity, sedentary lifestyles, and unhealthy dietary patterns (6, 7). These trends place increasing pressure on healthcare systems to deliver cost-effective, long-term disease management, highlighting the importance of efficiency in resource utilization. Understanding how effectively healthcare expenditure translates into population health outcomes is therefore increasingly important for healthcare policy and resource planning.

From a health economics perspective, evaluating system performance requires moving beyond single indicators such as expenditure levels or life expectancy. Efficiency analysis provides a more comprehensive framework by examining how well healthcare systems convert healthcare resources into population health outcomes (2, 8). In this context, Data Envelopment Analysis (DEA) has emerged as one of the most widely used methods for assessing relative efficiency across decision-making units.

Originally developed by Abraham Charnes, William *W. cooper*, and Edward Rhodes (9), DEA is a non-parametric linear programming technique that constructs an empirical efficiency frontier and evaluates the relative performance of each unit against best-performing peers. The method has been extensively applied in healthcare research, including hospital efficiency studies, primary care assessments, and cross-country health system comparisons (10–12). More recent studies have applied DEA to evaluate national health system efficiency across high and middle-income countries, demonstrating substantial variation in how effectively resources are translated into health outcomes (3, 13–15).

Despite the growing body of global literature, evidence on health system efficiency within the GCC region remains limited. Existing studies have primarily focused on individual countries or healthcare facilities within the GCC region, with relatively limited evidence directly comparing healthcare efficiency across GCC member states (16, 17). Given the similarities in economic structure, healthcare financing models, and disease burden across GCC countries, a focused comparative analysis offers valuable insights into relative performance and potential efficiency gains.

Several studies have applied DEA to evaluate healthcare system efficiency at national, regional, and international levels. These include cross-country analyses of OECD countries, Latin American and Caribbean countries, and Eastern Mediterranean countries (13, 15, 18). DEA has also been applied at the healthcare facility level within Saudi Arabia (17). A summary of selected country-level and regional studies is presented in Table 1.

**Table 1:** Summary of selected studies applying DEA to healthcare system efficiency assessment.

Study	Region	Sample size	Main findings
Varabyova and Schreyögg (13)	OECD countries	29 countries	Reported substantial variation in healthcare-sector efficiency across OECD countries, indicating that comparable economic development and healthcare investment do not necessarily produce similar efficiency levels.
Seddighi et al. (18)	Eastern Mediterranean Region	16 countries	Identified variation in health-system efficiency across Eastern Mediterranean countries using DEA, highlighting differences in how countries convert healthcare resources into population health outcomes.
Peres et al. (15)	Latin America and Caribbean	33 countries	Found considerable efficiency variation across Latin American and Caribbean healthcare systems, suggesting that healthcare expenditure alone does not explain differences in health outcomes.

As shown in Table 1, previous DEA studies have generally examined larger country samples across broader international or regional settings. In contrast, evidence directly comparing healthcare expenditure efficiency across the six GCC member states remains limited. Therefore, the present study provides an exploratory GCC-focused assessment of relative healthcare expenditure efficiency using a parsimonious DEA framework.

Furthermore, improving healthcare efficiency is a central objective of ongoing national reform initiatives, notably Saudi Arabia’s Vision 2030 Health Sector Transformation Program (19, 20). These reforms emphasize value-based care, strengthening primary healthcare, and optimizing resource allocation. Empirical evidence on efficiency is therefore essential to support policy decisions and guide resource prioritization.

This study aimed to provide an exploratory evaluation of healthcare expenditure efficiency across GCC countries using DEA. Specifically, the study examined how effectively GCC countries translate healthcare expenditure into population health outcomes, identified countries positioned on the efficiency frontier within the model, and explored potential differences in relative efficiency across healthcare systems. The findings were intended to provide preliminary evidence to support healthcare policy discussions and resource-allocation strategies within the GCC region.

## 2 Methods

### 2.1 Study design and analytical framework

This exploratory study employed a cross-sectional comparative design to evaluate the relative efficiency of healthcare expenditure across GCC countries using DEA. The analysis was grounded in the health production function framework, in which healthcare resources are transformed into population health outcomes (2, 8). Within this framework, efficiency refers to the extent to which healthcare systems are able to achieve favorable health outcomes relative to resource utilization (1, 2).

Because the number of decision-making units (DMUs) was limited to the six GCC countries, the DEA model was intentionally simplified to reduce dimensionality and improve discriminatory power. Commonly cited DEA guidelines recommend that the number of DMUs should be at least three times the combined number of input and output variables, or exceed the product of the number of inputs and outputs (21). Given the small number of available DMUs, the present study employed a parsimonious specification consisting of one input and one output variable. With six DMUs and two total variables, the model satisfies these commonly cited sample adequacy recommendations. Nevertheless, because DEA performance may be affected by small sample sizes, the analysis was explicitly framed as exploratory, and the findings should be interpreted cautiously.

### 2.2 Data sources

Data were obtained from internationally recognized databases, including the World Bank World Development Indicators (WDI), the World Health Organization (WHO) Global Health Observatory, and WHO Global Health Expenditure Database (4–6). These sources provide standardized and internationally comparable indicators suitable for cross-country efficiency analysis.

The exact values, data years, and indicator definitions used in the analysis are reported in Table 2.

**Table 2:** Health system inputs and outputs across GCC countries.

Country	Health exp per capita (PPP, USD) (2022)	Physicians per 1,000 population (latest available)	Hospital beds per 1,000 population (latest available)	Life expectancy at birth (years) (2022)	NCD mortality rate per 100,000 population (age-standardized, 2019)
Saudi Arabia	3,265	3.5	2.2	77.3	493
UAE	3,313	2.9	1.4	80.5	528
Qatar	2,469	2.5	1.3	81.9	650
Kuwait	2,294	2.3	2.0	80.6	324
Bahrain	2,370	0.7	1.7	81.0	644
Oman	1,301	2.0	1.5	77.9	684

Health expenditure per capita (PPP-adjusted, current international USD) and life expectancy at birth were obtained from the World Bank WDI for the year 2022. Additional descriptive indicators, including physician density, hospital bed density, and age-standardized NCD mortality rates, were collected to provide contextual interpretation of healthcare system characteristics across GCC countries.

Because complete data for all indicators were not available for a single common year across all countries, the most recent available observations were used while maintaining consistency in indicator definitions. This approach is commonly adopted in cross-country health system studies where perfect temporal alignment is not feasible (3, 13).

### 2.3 Variable selection

#### 2.3.1 Input variable

One input variable was selected:

- Health expenditure per capita (PPP-adjusted, USD)

Health expenditure per capita was selected because it represents the overall financial resources devoted to healthcare and is widely used in comparative health system efficiency analyses (2, 11, 12). The use of a single expenditure-based input also reduced model dimensionality, which is important in small-sample DEA applications.

#### 2.3.2 Output variable

One output variable was selected:

- Life expectancy at birth (years)

Life expectancy was selected as a broad and internationally comparable indicator of population health outcomes. It is commonly used in comparative health system evaluations because it reflects cumulative health system performance and overall population survival (2, 8).

Additional indicators, including physician density, hospital bed density, and NCD mortality rates, were retained in Table 2 for descriptive contextualization but were not included in the final DEA model to avoid excessive dimensionality and frontier inflation in the small DMU sample. Although healthcare system performance is inherently multidimensional, the present study adopted a parsimonious one-input, one-output specification because of the limited number of decision-making units available for analysis. Inclusion of additional inputs and outputs would have increased model dimensionality, reduced discriminatory power, and heightened the risk of frontier inflation in a small-sample DEA setting (21, 22). Health expenditure per capita was selected as a comprehensive measure of healthcare resource utilization, while life expectancy at birth was chosen as a widely used and internationally comparable indicator of population health outcomes.

### 2.4 Data envelopment analysis (DEA)

Technical efficiency was estimated using DEA, a non-parametric linear programming method originally developed by Charnes, Cooper, and Rhodes (CCR) (9) and subsequently extended to variable returns to scale (VRS) by Banker, Charnes, and Cooper (BCC) (10). DEA evaluates the relative efficiency of DMUs by constructing an empirical efficiency frontier from the best-performing units and comparing each DMU against this frontier. An efficiency score of 1 indicates that a DMU lies on the efficient frontier, whereas scores below 1 indicate relative inefficiency compared with peer units.

The input-oriented BCC model used in this study can be expressed as:

Minimize θ

Subject to:


∑λjxij≤θxio

∑λjyrj≥yro

∑λj=1

λj≥0

Where *θ* represents the technical efficiency score; λj denotes the intensity variables defining the reference set; xij represents input values; and yrj represents output values. The index *i* denotes the input, *r* denotes the output, *o* denotes the DMU under evaluation, and *j* denotes each DMU in the reference set. The convexity constraint ( ∑λj=1 ) allows for variable returns to scale and distinguishes the BCC model from the original CCR model (9, 10).

The VRS specification was selected to account for differences in country size and healthcare system scale across GCC countries. An input-oriented approach was adopted because the study aimed to assess the extent to which healthcare expenditure could theoretically be reduced while maintaining observed health outcomes, which is particularly relevant in policy contexts focused on healthcare resource optimization (2, 12).

A parsimonious one-input, one-output model specification was adopted to satisfy sample adequacy requirements and reduce the risk of frontier inflation associated with small samples. A commonly cited recommendation suggests that the number of DMUs should be at least three times the combined number of inputs and outputs (21).

Efficiency scores range from 0 to 1, where a score of 1 indicates frontier efficiency within the model and values below 1 indicate lower relative efficiency.

### 2.5 Data analysis

The dataset was structured with one observation per country. Prior to analysis, all variables were checked for unit consistency, completeness, plausibility, and agreement with original source databases. Variables identified during data verification as inconsistent with source datasets were corrected before final DEA estimation.

Given the limited number of countries included in the GCC sample, the DEA results were interpreted as exploratory. No inferential claims were made regarding statistically significant differences in efficiency scores.

DEA was conducted using R software (version 4.3.1) with the Benchmarking package. A VRS, input-oriented model was specified. Efficiency scores were calculated for each country, and countries were compared based on their relative expenditure-efficiency profiles. Descriptive statistics were additionally used to compare healthcare expenditure and population health outcomes across GCC countries (3).

## 3 Results

### 3.1 Descriptive analysis of healthcare expenditure and health outcomes

Table 2 summarizes the distribution of healthcare expenditure, healthcare system capacity indicators, and population health outcomes across the six GCC countries. Substantial variation was observed across countries, reflecting differences in healthcare resource allocation and health system characteristics within the region.

Health expenditure per capita (PPP-adjusted) ranged from approximately USD 1,301 in Oman to USD 3,313 in the United Arab Emirates. Physician density varied considerably, ranging from 0.7 physicians per 1,000 population in Bahrain to 3.5 physicians per 1,000 population in Saudi Arabia. Hospital bed density ranged from 1.3 beds per 1,000 population in Qatar to 2.2 beds per 1,000 population in Saudi Arabia.

In terms of population health outcomes, life expectancy at birth was highest in Qatar (81.9 years) and lowest in Saudi Arabia (77.3 years). Age-standardized NCD mortality rates also varied substantially across GCC countries, ranging from 324 deaths per 100,000 population in Kuwait to 684 deaths per 100,000 population in Oman. These findings highlight considerable heterogeneity in healthcare expenditure patterns, healthcare system capacity, and health outcomes across the GCC region (4–6).

### 3.2 Data envelopment analysis (DEA) efficiency scores

The results of the input-oriented VRS DEA model are presented in Table 3. The analysis demonstrated variation in relative healthcare expenditure efficiency across GCC countries, indicating differences in how effectively healthcare expenditure is translated into population health outcomes, as measured by life expectancy.

**Table 3:** Technical efficiency scores across GCC countries (input-oriented VRS DEA model).

Country	VRS efficiency score	Relative position	Potential input reduction (%)
Qatar	1.000	1	0%
Oman	1.000	1	0%
Bahrain	0.931	3	6.9%
Kuwait	0.911	4	8.9%
UAE	0.622	5	37.8%
Saudi Arabia	0.398	6	60.2%

Qatar and Oman were positioned on the efficiency frontier within the exploratory DEA model, each achieving a relative efficiency score of 1.00. These findings indicate that, within the model specification used, both countries achieved comparatively favorable life expectancy outcomes relative to healthcare expenditure levels.

Bahrain and Kuwait also demonstrated relatively high efficiency scores of 0.931 and 0.911, respectively, suggesting comparatively lower input-reduction potential within the model. In contrast, the United Arab Emirates and Saudi Arabia exhibited lower relative efficiency scores of 0.622 and 0.398, respectively. Within the assumptions of the input-oriented model, these findings suggest comparatively higher healthcare expenditure relative to observed life expectancy outcomes.

However, these efficiency scores should not be interpreted as direct measures of healthcare quality, healthcare system effectiveness, or overall health system performance. DEA evaluates relative efficiency within the selected sample and model specification, and the results indicate how effectively healthcare expenditure is translated into the chosen output measure relative to peer countries included in the analysis.

Because the analysis included only six DMUs, the results should be interpreted cautiously. The simplified one-input/one-output DEA specification was designed to improve model discrimination in a small-sample setting.

### 3.3 Comparative efficiency patterns

The DEA findings demonstrated notable variation in relative healthcare expenditure efficiency across GCC countries. Countries positioned closer to the efficiency frontier generally achieved more favorable life expectancy outcomes relative to healthcare expenditure levels, whereas countries with lower efficiency scores demonstrated comparatively higher expenditure relative to observed health outcomes.

Qatar and Oman achieved frontier efficiency despite substantial differences in healthcare expenditure levels, suggesting that higher expenditure alone does not necessarily correspond to greater relative efficiency. Oman, in particular, demonstrated frontier efficiency despite having the lowest healthcare expenditure per capita within the GCC sample, whereas the United Arab Emirates demonstrated lower relative efficiency despite the highest expenditure level. Similarly, Saudi Arabia demonstrated the lowest efficiency score within the model, reflecting comparatively high expenditure levels relative to life expectancy outcomes.

These findings reinforce the broader concept that healthcare system efficiency depends not only on the magnitude of healthcare spending, but also on how effectively financial resources are translated into population health outcomes (3, 13). However, because the present analysis used a simplified exploratory DEA model with a limited number of DMUs, the findings should be interpreted cautiously. Additional analyses incorporating larger datasets, longitudinal designs, or expanded country samples may provide more robust insights into healthcare expenditure efficiency across the GCC region.

### 3.4 Sensitivity analysis

To assess the robustness of the findings, additional DEA models were estimated using alternative specifications, including constant returns to scale (CRS), VRS, input-oriented, and output-oriented models. Scale efficiency scores were also calculated. The results are presented in Tables 4, 5.

**Table 4:** Sensitivity analysis of DEA efficiency scores across GCC countries.

Country	VRS-input	CRS-input	Scale efficiency	VRS-output	CRS-output
Saudi Arabia	0.398	0.395	0.992	0.944	0.395
UAE	0.622	0.406	0.653	0.983	0.406
Qatar	1.000	0.554	0.554	1.000	0.554
Kuwait	0.911	0.587	0.644	0.991	0.587
Bahrain	0.931	0.571	0.613	0.993	0.571
Oman	1.000	1.000	1.000	1.000	1.000

**Table 5:** Reference sets and benchmark peers.

Country	Reference set
Saudi Arabia	Oman
UAE	Qatar, Oman
Kuwait	Qatar, Oman
Bahrain	Qatar, Oman
Qatar	Frontier country
Oman	Frontier country

Oman remained efficient across all model specifications, while Saudi Arabia consistently exhibited the lowest efficiency scores. Although efficiency estimates varied under alternative assumptions, the overall pattern of relative performance remained broadly consistent. These findings suggest that the principal conclusions of the study were reasonably robust to alternative DEA specifications despite the limited number of decision-making units included in the analysis.

## 4 Discussion

### 4.1 Principal findings

This study provides an exploratory comparative assessment of healthcare expenditure efficiency across GCC countries using DEA. Qatar and Oman were positioned on the efficiency frontier, while Bahrain, Kuwait, the United Arab Emirates, and Saudi Arabia demonstrated comparatively lower scores, a pattern that held across alternative model specifications.

A central finding is that higher healthcare expenditure did not consistently correspond to more favorable population health outcomes. Some countries with lower spending achieved comparable or better life expectancy than countries allocating substantially greater resources, supporting the view that efficiency depends on how resources are used, not merely how much is spent (1, 2, 14).

The efficiency scores reported here reflect relative performance within this specific DEA framework. Countries with lower scores should not be read as having poor healthcare systems, nor should frontier countries be assumed to excel in all dimensions of performance. The results are bounded by the comparator group and the variables selected.

### 4.2 Drivers of efficiency differences

The observed variation in relative expenditure efficiency across GCC countries may reflect differences in healthcare system organization, resource allocation strategies, preventive healthcare approaches, and population health profiles rather than differences in expenditure levels alone.

Countries positioned on the exploratory efficiency frontier, particularly Qatar and Oman, demonstrated comparatively favorable life expectancy outcomes relative to healthcare expenditure levels within the DEA model. However, frontier positioning should not be interpreted as definitive evidence of superior healthcare system performance, particularly given the exploratory nature of the model and the limited number of DMUs included in the analysis. One possible explanation may relate to differences in preventive care, public health strategies, healthcare accessibility, and efficiency of resource utilization. Previous studies have suggested that health systems emphasizing preventive care and primary healthcare may achieve favorable outcomes at lower cost, supporting this interpretation (14, 15, 23).

In contrast, lower relative efficiency scores observed for the United Arab Emirates and Saudi Arabia may reflect comparatively high healthcare expenditure levels relative to observed life expectancy outcomes. These findings do not necessarily indicate poor healthcare quality, but rather suggest that higher expenditure alone does not automatically translate into proportionally better population health outcomes. Similar observations have been reported in international health system efficiency studies, where variation in efficiency persists even among countries with comparable economic status and healthcare spending levels (13, 15).

Bahrain and Kuwait demonstrated intermediate efficiency profiles within the model. Kuwait’s comparatively favorable NCD mortality profile and Bahrain’s relatively low physician density may partially contribute to their observed efficiency patterns, highlighting the influence of both expenditure levels and broader health system characteristics on DEA outcomes.

Interpretation of life expectancy outcomes within GCC countries should be approached cautiously because demographic structures differ substantially across the region, particularly with respect to expatriate populations. Countries such as Qatar and the United Arab Emirates have exceptionally large expatriate populations composed predominantly of younger working-age adults, resulting in lower dependency ratios and a demographic profile that differs markedly from that of countries with larger proportions of permanent national populations. This phenomenon is often described as a “healthy worker effect,” whereby healthier individuals are more likely to migrate for employment opportunities, potentially contributing to more favorable population health indicators.

Consequently, national life expectancy estimates in some GCC countries may partially reflect demographic composition rather than healthcare system performance alone. Because life expectancy was used as the sole output variable in the present DEA model, differences in demographic structure may have influenced the relative efficiency scores observed. Therefore, the frontier positioning of countries such as Qatar should not be interpreted exclusively as evidence of superior healthcare system performance. Future studies should consider incorporating age-standardized health indicators, demographic adjustment methods, or additional outcome measures to better account for population structure differences across GCC countries.

### 4.3 Policy implications for GCC health systems

The findings have preliminary implications for healthcare policy across GCC countries. The observed variation in expenditure efficiency suggests that improvements in population health outcomes may be achievable through more effective allocation and utilization of healthcare resources, rather than through expenditure increases alone.

These findings align with ongoing healthcare reform initiatives across the GCC region, including Saudi Arabia’s Vision 2030 Health Sector Transformation Program, which emphasizes value-based care, preventive medicine, healthcare system sustainability, and optimization of healthcare resource utilization (19, 20).

For Saudi Arabia specifically, the relatively lower efficiency score observed in the present model may suggest opportunities to strengthen preventive healthcare services, improve care coordination, and enhance long-term chronic disease management. Previous research has suggested that healthcare reforms emphasizing integrated care models, digital health systems, and primary healthcare strengthening may improve healthcare efficiency and system responsiveness (20, 24).

More broadly, GCC healthcare systems may benefit from policies focused on preventive care, public health interventions, healthcare system integration, and evidence-based resource allocation. These approaches are increasingly recognized as important components of efficient healthcare system performance (2, 3).

### 4.4 Comparison with previous literature

The findings of the present study are consistent with previous DEA-based evaluations of healthcare system efficiency. Varabyova and Schreyögg (13) reported substantial variation in healthcare efficiency across OECD countries despite comparable levels of economic development and healthcare investment. Peres et al. (15) observed considerable heterogeneity in healthcare system efficiency among Latin American and Caribbean countries, demonstrating that higher healthcare expenditure does not necessarily translate into proportionally better population health outcomes. In the Eastern Mediterranean context, Seddighi et al. (18) also reported variation in health-system efficiency, further supporting the observation that regional health systems differ in how effectively they convert healthcare resources into population health outcomes.

The present findings reinforce this observation within the GCC context. Although several GCC countries allocate substantial financial resources to healthcare, relative efficiency varied considerably across countries. In particular, Oman achieved frontier efficiency despite having the lowest healthcare expenditure per capita in the sample, whereas countries with higher expenditure levels did not necessarily demonstrate superior efficiency scores. These findings support previous evidence suggesting that healthcare efficiency depends not only on the amount of resources invested but also on how effectively those resources are translated into health outcomes (2, 3, 14).

Unlike many previous DEA studies that examined larger country samples or healthcare facilities, the present study focused exclusively on the six GCC member states. Consequently, the findings should be interpreted as an exploratory assessment of relative healthcare expenditure efficiency rather than definitive rankings of healthcare system performance. Nevertheless, the study contributes region-specific evidence to a relatively limited body of literature examining healthcare efficiency across GCC countries.

### 4.5 Strengths and limitations

This study has several strengths. It utilized standardized and internationally comparable data sources and applied DEA, a widely used method for comparative efficiency assessment. In addition, the focus on GCC countries provides region-specific insights that remain relatively underrepresented in the healthcare efficiency literature.

However, several limitations should be acknowledged. First, the analysis was based on aggregate national-level data, which may mask important within-country variations in healthcare access, service delivery, and population health outcomes. Second, differences in reporting years across indicators may introduce minor inconsistencies, although this approach is commonly adopted in cross-country comparative analyses when perfectly aligned data are unavailable (3).

Third, the DEA model used a limited number of DMUs (*n* = 6), which restricts discriminatory power and increases the risk of frontier inflation (21). To partially address this limitation, the final model was intentionally simplified to include a single expenditure input and a single population health output. Nevertheless, the findings should be interpreted as exploratory rather than definitive efficiency rankings (21, 22).

Also, DEA results are sensitive to variable selection and model specification. The simplified expenditure-life expectancy framework used in the present study does not fully capture the multidimensional complexity of healthcare system performance, and additional variables such as healthcare quality, service utilization, and disease-specific outcomes may influence efficiency patterns.

Sensitivity analyses using alternative DEA specifications demonstrated that the overall efficiency pattern remained broadly consistent. Oman remained efficient across all model specifications, whereas Saudi Arabia consistently exhibited the lowest efficiency scores. These findings provide additional support for the robustness of the exploratory results despite the limited number of decision-making units included in the analysis.

Although bootstrap DEA methods have been proposed to assess the statistical uncertainty of efficiency estimates, bootstrap procedures were not performed in the present study because the analysis was limited to six decision-making units. In very small DEA samples, bootstrap resampling may produce unstable interval estimates and should therefore be interpreted cautiously. Instead, robustness was assessed through sensitivity analyses using alternative DEA specifications, including CRS, VRS, input-oriented, and output-oriented models.

Finally, DEA measures relative technical efficiency rather than healthcare quality, healthcare effectiveness, or overall health system performance. The efficiency scores reported in this study are dependent on the selected input and output variables and the comparator countries included in the model. Therefore, the findings should be interpreted as indicators of relative healthcare expenditure efficiency rather than comprehensive evaluations of national healthcare systems.

### 4.6 Future research directions

Future research should incorporate larger datasets, longitudinal designs, or panel DEA approaches to improve model discrimination and robustness. Expanding the analysis to include additional countries or multiple years may provide more stable efficiency estimates and allow evaluation of temporal trends in healthcare expenditure efficiency across the GCC region.

Subnational analyses within countries such as Saudi Arabia may also provide more detailed insights into regional variation in healthcare expenditure efficiency. In addition, future studies should consider incorporating broader healthcare system indicators, including healthcare quality measures, healthcare utilization patterns, patient-reported outcomes, and disease-specific indicators, to provide a more comprehensive evaluation of healthcare system performance (2, 22).

## 5 Conclusion

GCC countries vary in how effectively they translate healthcare expenditure into population health outcomes. Qatar and Oman achieved frontier efficiency within the model, while the remaining four countries, Bahrain, Kuwait, the UAE, and Saudi Arabia, showed room for improvement relative to their peers. Notably, Oman reached the frontier with the lowest per capita spending in the sample, while Saudi Arabia and the UAE, despite the highest expenditure levels, scored lowest on efficiency.

These results carry practical relevance for ongoing reform efforts across the region, particularly initiatives focused on value-based care and resource optimization. They do not, however, constitute definitive rankings of healthcare system quality. The analysis is exploratory, bounded by six countries and a parsimonious model, and should be treated accordingly.

More future work with incorporating additional health system indicators, longer time horizons, and subnational data, would strengthen the evidence base and allow firmer conclusions about where and how efficiency gains are achievable across the GCC.

## Data Availability

The raw data supporting the conclusions of this article will be made available by the authors, without undue reservation.
